# Evolution of a Bidirectional Pediatric Critical Care Educational Partnership in a Resource-Limited Setting

**DOI:** 10.3389/fped.2021.738975

**Published:** 2021-10-15

**Authors:** Sarah E. Gardner Yelton, Julia M. McCaw, Carolyn J. Reuland, Diana A. Steppan, Paula Pilar G. Evangelista, Nicole A. Shilkofski

**Affiliations:** ^1^Department of Anesthesiology and Critical Care Medicine, Charlotte R. Bloomberg Children's Center, Johns Hopkins University School of Medicine, Baltimore, MD, United States; ^2^Johns Hopkins University School of Medicine, Baltimore, MD, United States; ^3^Department of Pediatric Critical Care Medicine, Philippine Children's Medical Center, Quezon City, Philippines; ^4^Department of Pediatrics, Charlotte R. Bloomberg Children's Center, Johns Hopkins University School of Medicine, Baltimore, MD, United States

**Keywords:** children, simulation, medical education, global health, program development

## Abstract

**Introduction:** Children in resource-limited settings are disproportionately affected by common childhood illnesses, resulting in high rates of mortality. A major barrier to improving child health in such regions is limited pediatric-specific training, particularly in the care of children with critical illness. While global health rotations for trainees from North America and Europe have become commonplace, residency and fellowship programs struggle to ensure that these rotations are mutually beneficial and do not place an undue burden on host countries. We created a bidirectional, multimodal educational program between trainees in Manila, Philippines, and Baltimore, Maryland, United States, to improve the longitudinal educational experience for all participants.

**Program Components:** Based on stakeholder input and a needs assessment, we established a global health training program in which pediatricians from the Philippines traveled to the United States for observerships, and pediatric residents from a tertiary care center in Baltimore traveled to Manila. Additionally, we created and implemented a contextualized simulation-based shock curriculum for pediatric trainees in Manila that can be disseminated locally. This bidirectional program was adapted to include telemedicine and regularly scheduled “virtual rounds” and educational case conferences during the COVID-19 pandemic. Providers from the two institutions have collaborated on educational and clinical research projects, offering opportunities for resource sharing, bidirectional professional development, and institutional improvements.

**Conclusion:** Although creating a mutually beneficial global health partnership requires careful planning and investment over time, establishment of a successful bidirectional educational and professional development program in a limited-resource setting is feasible and benefits learners in both countries.

## Introduction

Children in resource-limited settings (RLS) are disproportionately affected by common childhood illnesses, resulting in significant morbidity and mortality (1). Each year, 60,000 children under the age of 5 die in the Philippines, almost a third from sepsis, diarrhea, or pneumonia (1). Providers in RLS frequently need to resuscitate children with critical illness or life-threatening disease processes, but encounter obstacles that include limited pediatric-specific training, inadequate staffing, and equipment and financial constraints (2, 3). Although decreasing child mortality in RLS will require improved training, particularly in the areas of pediatric emergency medicine and critical care, the methods used to effectively enhance education are neither clear nor standardized (3–7).

Various methods to address these educational gaps have historically included (1) observerships in tertiary care hospitals, wherein individuals observe clinical care and participate in didactic education; (2) limited in-country educational programs; (3) telemedicine and telemonitoring programs; and 4) longer-term formalized training programs. Each of these methods has a unique set of barriers. For example, observerships are expensive and do not provide hands-on opportunities (8), and in-country educational efforts face challenges in sustainability, dissemination, and skill-decay (9). At the same time, global health rotations for trainees from North America and Europe have become commonplace, benefitting learners by exposing them to global disease burden, challenges in resource utilization, and different cultural belief systems that influence clinical practice (10). However, residency and fellowship training programs struggle to ensure that these rotations are mutually beneficial; they require careful design, implementation, and ongoing evaluation (10, 11).

Beyond the need to improve pediatric critical care training programs, the World Health Organization (WHO) and experts in the field of pediatric critical care in global health have identified several major target areas that could contribute to better provision of pediatric critical care and decrease child mortality. Examples include standardization of care, such as with the Surviving Sepsis Guidelines and the WHO guidelines for emergency treatment of children, care checklists, and early identification of decompensation both pre-hospital and in-hospital (4, 5, 7, 12, 13).

Using the above framework, we sought to create a long-term bidirectional, multimodal educational program between trainees in Manila, Philippines, and those in Baltimore, Maryland, United States (U.S.), using cross-institutional trainee experiences, formal simulation education development, and a collaborative research relationship. Through this partnership, we aim to improve the longitudinal educational experience for all participants and serve as a model for pediatric critical care education in other venues.

## Program Components

We have sustained a relationship between pediatric providers at Johns Hopkins Hospital (JHH) in Baltimore and Philippine Children's Medical Center (PCMC) in Manila for over 30 years. This relationship started with an observership program in which pediatricians from PCMC rotated in the pediatric intensive care unit (PICU) at JHH. The strong relationship that developed over time between the two institutions fostered the creation of a mutually beneficial partnership, which included ongoing needs assessments and programmatic changes to best focus on WHO-identified areas for improving provision of pediatric critical care.

### Observership

In 1996, before the establishment of formal pediatric critical care training in the Philippines, faculty from JHH and PCMC collaboratively created an observership program for pediatricians from PCMC interested in additional pediatric critical care training. Each year, two to three pediatricians from PCMC volunteered to participate in a program for several months at JHH. After the development of an accredited pediatric critical care fellowship training program in the Philippines, the senior pediatric critical care fellows from PCMC began traveling to Baltimore in their final year of training to participate in the observership. The 4–8-week-long program includes participation in PICU rounds and all didactic and simulation-based education available to faculty and trainees in the JHH PICU. There is no institutional funding available for this program, so participants are responsible for their own travel expenses.

Since the inception of the program, Johns Hopkins Pediatrics has received over 30 trainees (primarily PICU fellows). 15 of these participants currently hold leadership positions in pediatrics and pediatric critical care across the Philippines as program directors, unit medical directors, committee chairs and division heads. For example, one former trainee is currently head of the PICU at PCMC, serves as director of research and research training at her institution, in addition to maintaining a role in medical education as an assistant program director for the pediatric critical care fellowship. Multiple participants are also active in their national organizations in the Philippines Pediatric Society and the Society of Pediatric Critical Care Medicine, Philippines.

### Education/Simulation

Although observerships are a common method of education in global health, and participants report them to be enjoyable experiences that contribute to professional development, these programs are expensive and inherently lack hands-on opportunities owing to legal and licensure constraints of the U.S. healthcare system (8, 14). It is therefore essential to augment these programs through additional training.

To align with the WHO goal of care standardization and early identification of in-hospital decompensation (5, 7, 13) and to expand access to hands-on training, we created and implemented a contextualized, sustainable simulation-based shock curriculum for pediatric trainees in Manila that can be disseminated locally. This was undertaken as a pilot for a larger scale simulation education program. Simulation programs have been shown to effectively educate individuals in many countries in areas ranging from fluid management for patients in shock to cardiopulmonary resuscitation, improving participant confidence and knowledge (15–17). Greater confidence in skills and improved knowledge scores can translate into changes in clinical practice, although the effect on outcomes is less well-known (18).

We carried out a needs assessment via in-country meetings with the PCMC pediatric residency program director, pediatric critical care faculty and trainees, and hospital administration, and conducted in person observations in the PICU at PCMC. Hands-on simulation experience with patient management was identified as a priority need for pediatric trainees. We created and implemented a simulation-based shock curriculum by administering a half-day workshop at PCMC with 24 pediatric residents in March 2020. Collaboratively, we chose to focus on shock due to the high contribution of dengue shock, septic shock, and hypovolemic shock to mortality in the Philippines, and early recognition and appropriate management can drastically improve patient outcomes (19). The curriculum included a didactic portion, skills-based stations, and simulation scenarios/objective standardized clinical examinations. After the workshop, confidence in shock concepts and skills and performance on a simulation scenario as measured via a checklist improved significantly for the pediatric residents. The program, created and taught collaboratively by pediatric critical care faculty and fellows from both institutions, was designed to establish a low-cost and accessible model of simulation education in an RLS. Most of the materials and task-trainers used were brought from the U.S. and donated to PCMC. Additionally, PCMC faculty participation in the creation and teaching of the curriculum allowed for these individuals to become master trainers. Together with the donated materials, this served to promote sustainability and dissemination of the curriculum and to allow for provision of frequent refresher training sessions to mitigate skill decay.

### Global Health Rotation

To maintain a bidirectional relationship, we also established a global health training program and rotation for pediatric residents from JHH in 2018. Our goal was to obtain maximal educational value for pediatric trainees from the U.S. while allowing for benefit to the host country. Essential components of a successful structured global health rotation include pre-departure orientation and simulation sessions focusing on local epidemiology and disease burden, early establishment of host-country needs and knowledge gaps, on-site mentorship with ongoing program evaluation, and a partnership with formalized longitudinal education and opportunities for professional development (6, 7, 10, 11, 20).

Our global health rotation adheres to these principles through a structured elective rotation in global health for JHH pediatric residents. Therefore, our program incorporates pre-departure training, pediatric training program leadership commitment to global health, and bilateral evaluations of the established relationship between PCMC and JHH. Global health elective rotations, with defined goals and objectives, are used to provide structured education and orientation for JHH residents before and during travel to the Philippines. The elective starts with a pre-departure curriculum and orientation that uses components of the Simulation Use for Global Away Rotations (SUGAR) curriculum, which includes simulations and procedure adaptations for limited-resource settings (21). The pre-departure curriculum also includes lectures on bioethics, professionalism, and Filipino culture from pediatric faculty with experience in global health. While in the Philippines, visiting JHH residents spend 1–2 days observing rounds in each unit at PCMC (PICU, neonatal intensive care unit, emergency department, and general ward); anesthesia-trained residents also have the opportunity to observe in the operating room. PCMC attendings, as well as JHH attendings traveling with residents during the elective abroad, give didactics, specifically case presentations with review of tropical and endemic diseases. PCMC has received 18 residents, fellows and medical students for observerships from Johns Hopkins University School of Medicine (JHUSOM).

Additionally, pediatric residents participating in the global health rotation travel to other regions of the Philippines to teach the simulation-based training programs Helping Babies Breathe (HBB) and Helping Mothers Survive (HMS). The team from JHUSOM has trained 65 midwives, nurses, physicians and nurse midwives as master trainers. The U.S.-based training team and the local master trainers have subsequently trained over 550 nursing students, nurses, midwives, physicians, and emergency first responders as providers in both training programs over the last 5 years. While much of the dissemination work has been focused in the Southern Philippines regions of Mindanao where neonatal and maternal mortality rates are highest in the country, both simulation-based training programs have been disseminated by trainers within Luzon, Visayas and Mindanao, stretching across many regions and various islands in the Philippines, with training materials having been translated into local dialects in these regions by bilingual master trainers.

Given the natural turnover of pediatric residents, relationships between faculty at JHH and PCMC have been key in establishing continuity and sustainability. The JHH pediatric residency director (NAS) has a longstanding relationship with many physicians at PCMC and has personally accompanied groups of residents to PCMC at the start of their global health rotation there. Hence, she has been able to continually assess host-country needs and knowledge gaps of U.S.-based residents, while providing mentorship for residents both in-country and upon return to the U.S. Additional programmatic assessment occurs via post-rotation evaluations by JHH residents on their return to the U.S.

American trainees who have participated in the global health rotation more recently have gone on to fellowship training in specialty fields within pediatrics and several are now in academic positions at major teaching hospitals in the United States, with continued academic focus in global health research and education. Many former trainees from both PCMC and JHUSOM have cited their experience on rotations resulting from the educational exchange as being very influential in their long-term career development, having been a major contributor to their understanding of other healthcare systems within global health settings. One of the trainees from Johns Hopkins stated: “Participating in this program appealed to me due to its sustainability. The opportunity to work with both community providers and specialists during a global health rotation and to experience disparities in care in different settings was part of the draw of the rotation, and something that has led me to pursue additional work in international settings in order to better understand disparities in healthcare in my own setting and practice. The idea that you are starting a cascade of education that is going to continue far beyond your time in the country is really exciting. The experience will also help me in my future career plans to practice pediatrics in austere settings where resources will be limited.”

### Research/Professional Development

Finally, providers from the two institutions have collaborated on clinical and educational research projects that have permitted opportunities for bidirectional professional development. This concept of “participatory research,” whereby the host country identifies specific research needs, and is partnered with visiting trainees whose skills and interests fill those needs, can lead not only to sustainable community health and hospital initiatives, but also to further understanding of care delivery needs in RLS for both local governments and global health practitioners (20). Additionally, it provides an opportunity for providers from both countries to present together at conferences, co-write manuscripts, and share resources (10, 11).

As previously mentioned, our research collaborative evaluated the efficacy of the simulation-based shock curriculum on skill and knowledge development. After participation in the curriculum, residents improved with respect to confidence and simulated performance in skills associated with shock identification and management. Simulation performance was measured using an original checklist, created based on previously validated checklists (22–24). While knowledge scores did not improve on a written assessment, checklist scores improved significantly following the intervention. Checklists are commonly used in simulation studies as a marker of knowledge acquisition, and may translate more directly to clinical skills than a written evaluation (22–25).

Additionally, the research collaborative evaluated the barriers and facilitators to implementation of a Pediatric Early Warning Score (PEWS) system at PCMC. Globally, pediatric hospitals have implemented PEWS systems to improve early detection of clinical deterioration in pediatric patients by standardizing escalation of care decisions. These scoring systems include various vital signs and other clinical characteristics for quick classification of decompensation risk. They have been shown to accurately predict the need for intensive care unit level of care and are better predictors of clinical deterioration than physician opinion alone (26–34). Implementation studies have shown that clinical outcomes are improved in some settings because the early recognition of patient decline allows for earlier intervention (34). PEWS systems have been modified to fit specific hospital contexts worldwide, including in some RLS (26–28, 30, 35).

We conducted semi-structured interviews with nurses, residents, fellows, and attendings at PCMC to characterize existing systems for escalation of care decisions and attitudes about PEWS implementation. In-person hospital observations by the study team at PCMC served to triangulate interview findings. Barriers within the PCMC workflow included limited bed capacity, delay in referral owing to uncertainty of patient condition severity, patient overflow, limited monitoring equipment, and high patient-to-staff ratio. Facilitators of PEWS implementation included positive attitudes toward PEWS adoption/adaptation and existence of systems for vital sign monitoring across different units. This study showed that tools such as the PEWS system may be feasible for implementation in RLS, and we anticipate that this formal assessment will result in PEWS system implementation at PCMC.

Pediatric faculty and trainees from both JHH and PCMC participated in the creation and implementation of both projects, and resources such as statistical support, editing services, and funding for manuscript submission were shared. The results of collaborative projects have been presented at the International Pediatric Simulation Society Virtual Workshop, the World Federation of Pediatric Intensive & Critical Care Societies (WFPICCS) Virtual Conference, and the Society for Critical Care Medicine Annual Congress. Additionally, manuscripts have been submitted for publication, allowing for multi-institutional authorship and bidirectional networking opportunities (36).

Distance mentorship has been a core component of the bidirectional exchange for several years, both in areas of clinical practice and within research. Specialists in both institutions have spoken and presented posters and abstracts together at national and international conferences on various topics in pediatric critical care medicine. Difficult case presentations and clinical conundrums have been discussed between the care teams in both institutions, as a form of distance mentoring for both fellows and early career faculty. Research mentorship in study design/execution and research ethics from faculty in both institutions has been a core tenet of all collaborative research and quality improvement endeavors undertaken by the two institutions, with input from institutional review boards in both countries. These opportunities have allowed, for example, one pediatric critical care attending to participate in the quality improvement process at her home institution through the PEWS project, attend WFPICCs and serve as a senior author on three oral presentations at the conference, in addition to authoring multiple manuscripts currently submitted for publication in major medical journals.

## Challenges

### COVID-19 Pandemic

When the COVID-19 pandemic restricted travel, both the observership program and the global health rotation required substantial adaptation. Medical education became increasingly provided via telemedicine throughout the pandemic (37), and we began using this technology in the fall of 2020 to sustain the program. Participants from PCMC were invited to attend regularly scheduled virtual PICU rounds and educational conferences remotely. To facilitate proper social distancing at JHH, both rounds and conferences were already conducted in a remote fashion through videoconferencing software using a tablet on wheels with video and microphone capabilities. This setup not only allowed for continued implementation of the observership in a virtual format, but also decreased costs associated with an in-person observership, while expanding access in an interdisciplinary/interprofessional fashion to other individuals at PCMC, including nursing and respiratory therapy.

When residents and fellows could no longer travel to Manila, pre-departure lectures were converted to a virtual format and expanded to include topics such as disaster medicine and tropical disease identification and management. SUGAR simulations were also adapted for appropriate social distancing and cleaning between groups. In addition to the development of the remote observership as described above, the 2-week-long global health rotation also featured shared synchronous lectures and case discussions between the global health residents and PCMC residents. The ability to adapt the curriculum to a virtual format and schedule shared lectures between programs—even when in-person observerships are not possible—highlights the positive relationship between JHH and PCMC and the sustainability of this partnership.

### Research Approval

We have struggled throughout the development of this program with timely implementation, in addition to inclusion of non-physician disciplines. The reasons for this are multifactorial, including cultural differences in communication styles, and lack of familiarity with institutional approval processes. While U.S. physicians are highly reliant on e-mail communication, this is not the case in the Philippines. Through early visits to PCMC, we were able to find alternative and more efficient methods of communication to improve collaboration, such as via phone messenger applications. The approval process commonly required in-person meetings or provision of hard-copies of paperwork. Travel to the country prior to implementation of the project was therefore essential to meet face-to-face with administration for a clear understanding of the process, find meeting proxies, and determine how best to transfer required paperwork.

Additionally, the research approval process to enroll physicians versus non-physician disciplines was different. While we were able to interview nurses for the PEWS project, we did not have approval to enroll nurses in our simulation-based education program in time for implementation. Future visits should include a focus on meeting with nursing leadership and a better understanding of the best process for nursing involvement, as inclusion of nursing in educational or clinical interventions is essential for meaningful and long-term change.

## Discussion

Establishing a mutually beneficial relationship between an institution in a resource rich setting and one in a RLS requires time, commitment, and stakeholder investment. We have been successful in sustaining a multimodal partnership via cross-institutional trainee experiences, simulation education development, and collaborative research. This partnership may serve as a model for educational programs in other RLS.

Although observerships are not the most effective method of education, we have endeavored to make them more fruitful by improving accessibility through telemedicine and adding hands-on education via simulation. Our resident global health elective adheres to ethical standards with ongoing reassessment that benefits both institutions. We have laid the groundwork for continued simulation education at PCMC that could be elaborated upon to encompass other topics and scenarios beyond shock in a contextualized manner.

Individuals from both institutions have benefited from resource-sharing in the realm of professional development via international conferences, publications, and participatory research on topics that address WHO goals of early identification of decompensation and standardization of care, as well as stakeholder-identified topics such as implementation of a simulation-based education program or PEWS system. Local identification of education, research, and improvement projects have contributed to sustainability because of buy-in from hospital healthcare providers and leadership.

PCMC is a large academic institution with a pediatric critical care training program, resources such as ventilators and sub-specialists, and trainees who are comfortable speaking English in medically complex situations. The generalizability of this program is limited to other regions or countries with similar resources and epidemiology of disease burden. Additionally, a relationship such as this has been fostered over decades of modification. Although it would likely present different challenges, implementing a similar design in another environment would be feasible and likely benefit from a similar framework that includes ongoing needs assessments with stakeholder buy-in, longitudinal educational programs, structured global health experiences and observerships, and a research collaborative that fosters professional development. Our ability to transition many educational components to virtual platforms may allow us to further expand program access to other institutions in the future. However, the in-person experiences remain essential, and must be reincorporated as international circumstances allow.

Additionally, though portions of the program have been evaluated formally, such as the simulation program and the global health rotation, most markers of our success are subjective or anecdotal, in the form of perceived improved relationships across institutions and positive verbal feedback. To best determine next steps for this program, a more formal assessment should be undertaken. Monitoring clinical improvement in patient outcomes over time would be beneficial. However, advances in medicine and variable availability of technologies, staffing, or funding serve as confounding factors that decrease the validity of outcomes as markers of programmatic success.

By adhering to guidance from the WHO and focusing on improving training in pediatric critical care, we aim to decrease obstacles associated with the health care of children with critical illness and the associated morbidity. We have built a successful collaboration between physicians across institutions who continue to assess the relationship and identify areas for improvement. Changes in clinical outcomes will require widespread and long-term use of an educational program that comes from local stakeholder commitment and institutional buy-in.

## Author's Note

The senior author NS has received research funding from the U.S. Department of Education Fulbright-Hays Group Projects Abroad Program and from the Laerdal Foundation to support work that is unrelated to the research outlined in the manuscript. These funding sources did not provide any support for the conduct of the research described herein.

## Data Availability Statement

The raw data supporting the conclusions of this article will be made available by the authors, without undue reservation.

## Ethics Statement

The studies involving human participants were reviewed and approved by the Johns Hopkins Hospital IRB and the Philippine Children's Medical Center Office of Research Development. The patients/participants provided their written informed consent to participate in this study.

## Author Contributions

All authors listed have made a substantial, direct and intellectual contribution to the work, and approved it for publication.

## Funding

Investigators SG and JM are recipients of the Lietman Travel Award from the Johns Hopkins Center for Global Health, which funded travel and selected educational materials. This funding source provided support for conduct of the research but did not play a role in the study design, data collection, analysis, or decision to submit the paper for publication.

## Conflict of Interest

The authors declare that the research was conducted in the absence of any commercial or financial relationships that could be construed as a potential conflict of interest.

## Publisher's Note

All claims expressed in this article are solely those of the authors and do not necessarily represent those of their affiliated organizations, or those of the publisher, the editors and the reviewers. Any product that may be evaluated in this article, or claim that may be made by its manufacturer, is not guaranteed or endorsed by the publisher.
